# Deep Ensembling of Multiband Images for Earth Remote Sensing and Foramnifera Data

**DOI:** 10.3390/s25072231

**Published:** 2025-04-02

**Authors:** Loris Nanni, Sheryl Brahnam, Matteo Ruta, Daniele Fabris, Martina Boscolo Bacheto, Tommaso Milanello

**Affiliations:** 1 Department of Information Engineering, University of Padova, 35139 Padova, Italy; matteo.ruta@studenti.unipd.it (M.R.); daniele.fabris.5@studenti.unipd.it (D.F.); martina.boscolobacheto@studenti.unipd.it (M.B.B.); tommaso.milanello@studenti.unipd.it (T.M.); 2 Information Technology and Cybersecurity, Missouri State University, 901 S. National, Springfield, MO 65804, USA; sbrahnam@missouristate.edu

**Keywords:** convolutional neural network, ensemble learning, image classification, multichannel image, satellite images

## Abstract

The classification of multiband images captured by advanced sensors, such as satellite-mounted imaging systems, is a critical task in remote sensing and environmental monitoring. These sensors provide high-dimensional data that encapsulate a wealth of spectral and spatial information, enabling detailed analyses of the Earth’s surface features. However, the complexity of these data poses significant challenges for accurate and efficient classification. Our study describes and highlights methods for creating ensembles of neural networks for handling multiband images. Two applications are illustrated in this work: (1) satellite image classification tested on the EuroSAT and LCZ42 datasets and (2) a species-level identification of planktic foraminifera. Multichannel images are fed into an ensemble of Convolutional Neural Networks (CNNs) (ResNet50, MobileNetV2, and DenseNet201), where each network is trained using three channels obtained from the multichannel images, and two custom networks (one based on ResNet50 and the other one based on attention) where the input is a multiband image. The ensemble learning framework harnesses these variations to improve classification accuracy, surpassing other advanced methods. The proposed system, implemented in MATLAB 2024b and PyTorch 2.6, is shown to achieve higher classification accuracy than those of human experts for species-level identification of planktic foraminifera (>92% vs. 83%) and state-of-the-art performance on the tested planktic foraminifera, the EuroSAT and LCZ42 datasets.

## 1. Introduction

Multiband images are characterized by their representation of data across multiple spectral or feature dimensions. Multiband images are increasingly prevalent in various fields of study. Unlike traditional grayscale or RGB images, multiband images provide a richer and more detailed view of the underlying data, capturing diverse information that spans beyond the visible spectrum or typical feature space. The added complexity of multiband images opens avenues for advanced analysis, including precise classification tasks in domains such as remote sensing, medical imaging, industrial inspection, and scientific research [1].

The classification of multiband images poses unique challenges and opportunities. The high dimensionality of the data often encapsulates critical features necessary for distinguishing classes, yet it also introduces noise, redundancy, and computational complexity. To address these challenges, researchers have developed a range of techniques for feature extraction, dimensionality reduction, and classification that aim to balance accuracy and efficiency.

Computational methodologies for multiband image classification include both traditional machine learning and neural network approaches. Traditional machine learners, whether supervised or unsupervised, rely on linear or nonlinear transformations to extract relevant and intrinsic features [2]. Principal Component Analysis (PCA) is frequently employed as a transformation technique to reduce dimensionality while preserving essential variance, making it a common preprocessing step in classic machine learning and pattern recognition. A drawback of PCA is that it assumes linearity in the data, which can lead to the loss of important nonlinear relationships present in the original feature space. In [2], the authors proposed three PCA variants for multiband image classification: KPCA, which resulted in an overall accuracy (OA) of 95.92, KECA (95.63), and FPCA (95.13). The latter, while exhibiting lower performance than the other two PCA methods, had the advantage of providing less space complexity. Computational inefficiency is commonplace with multiband image classification. In [3], the authors proposed combining PCA and local binary patterns (LBPs) to address this problem. In [4], a local neighborhood structure preserving embedding that combined former label information produced an OA of 94.23.

Subpixel component analysis (SCA), proposed in [5], produced a high-performance OA of 97.79. In [6], the authors devised a method to automatically determine the optimal number of superpixels for multiband image classification; this determination resulted in an OA of 95.95. In [7], a tree-based classifier was proposed that addressed the limitation of PCA to handle only linear datasets; this method obtained an OA of 97.93. Gradient boosting decision tree regression was applied in [8], resulting in an OA of 97.00. Many authors have focused only on spectral data. The work in [9] recorded exceptional OA performance (99.80) by including both spectral and spatial information with tree-based models.

In terms of classifiers, several K-means methods have been examined to classify multiband images [10,11,12]. In [12], for instance, this approach resulted in an OA of 92.70. K-means performance has generally proven weak. Support Vector Machines (SVMs) are high-performing classifiers known for their effectiveness in handling high-dimensional data and complex decision boundaries. Papers using support vector machines (SVMs) include [13,14,15]. In [13], simple operations on multiband images obtained an OA of 99.73. In [14,15], which incorporated both spectral and spatial information in the images, the respective authors reported an OA of 95.24 and 96.74. Of note is that the authors in [14] combined a genetic algorithm with SVM.

Ensembling is a technique in machine learning that combines multiple models to improve overall performance, robustness, and generalization. An ensemble combines multiple models to improve accuracy, reduce overfitting, and enhance generalization. Ensembles take advantage of the weaknesses in individual models, thereby reducing variance and bias while enhancing predictive accuracy. A popular ensemble learning method is Random Forest (RF), which constructs multiple decision trees using random subsets of data and features. Exponentially weighted RF for multiband classification was examined in [16]; it produced an OA of 95.01. In [17], the authors compared different traditional classifiers (SVM, KNN, DT, and RF); PCA and minimum noise fraction (MNF) were used as transformers to minimize noise. RF was shown to be the most computationally efficient and produced the best OA of 99.65. A robust and efficient classification methodology that combined PCA, Local Binary Pattern (LBP), and the Back Propagation Neural Network (BPNN) was introduced in [18].

Engineered feature extraction, e.g., PCA and LBP, is not always necessary with neural networks (NNs). Approaches based on NNs can be divided into traditional neural networks and deep learning. Deep learning (DL) is a subset of neural networks that utilizes multiple hidden layers to automatically learn hierarchical feature representations, whereas traditional neural networks typically have fewer layers and rely more on manually engineered features. Image classification has seen dramatic advancements with the rise in DL, the most famous arguably being Convolutional Neural Networks (CNNs). They have consistently demonstrated superior performance in various image classification challenges [19,20] and have been shown in a number of studies to work well with multiband image classification, as seen in the following (reference—OA): Ref. [21]—98.09; Ref. [22]—96.00; Ref. [23]—99.3 (using real-time data); Ref. [24]—98.00; Ref. [25]—99.17; and Ref. [26]—99.94. Some problems with CNNs are that they struggle with capturing long-range dependencies due to their localized receptive fields and are limited in their ability to model complex spatial relationships without additional mechanisms such as attention or recurrence. Some attention-based networks include [27], which resulted in an OA of 90.91, and an attention-based domain adaptation using a residual network [28], with an OA of 97.97. CNN features can also be extracted and used with traditional classifiers and in ensembles, as in [29], which produced an OA of 97.47.

The accuracy of multiband image classification methods varies widely depending on the dataset and test protocol. One challenge in this field is the frequent use of different datasets with varying evaluation protocols. The results can vary significantly due to the lack of a standardized protocol for validating multiband classification systems. Fortunately, some datasets, such as those used in this paper, have recently become available that provide a clear protocol, including one that has recently been used in several papers, making a fair comparison possible.

This paper explores a practical application of ensemble learning (EL) on two classification tasks using these newer datasets. The first task addresses the problem discussed in [30], which involves training a CNN for foraminifera classification—a topic of significant interest in both industrial and research contexts [31]. Planktic foraminifera species serve as paleo-environmental bioindicators, with their radiocarbon measurements providing insights into parameters such as global ice volume, temperature, salinity, pH, and nutrient levels in ancient marine environments. Traditionally, foraminifera classification is carried out by large groups of human experts. This process is repetitive, labor intensive, and time consuming. Since the early 1990s, several efforts have aimed to automate this task [32]. Despite notable advancements, most methods still required substantial human oversight. However, the development of the neural network SYRACO2 in 2004 marked a significant breakthrough by reliably automating the identification of single-celled organisms [33]. In 2017, CNNs demonstrated substantial success in diatom identification [34], suggesting that further advancements in CNN technology could effectively alleviate the demanding task of foraminifera classification. In [30], the authors utilized a combination of ResNet50 and Vgg16, employing colorization techniques based on intensity percentiles across sixteen grayscale channels. Several novel colorization methods are introduced in [35] that diverge from the current state-of-the-art approaches [36] yet deliver impressive ensemble results. The findings in [35] demonstrated that training diverse CNN models on differently colorized images enhances system performance compared to other leading methods. Impressively, in both of these studies and in [30], the proposed systems surpassed human experts in classification accuracy.

The second classification task is satellite image classification; for this task, we have assessed performance using two datasets: EuroSAT and LCZ42. This task is related to the classification of land use and land cover (LULC), both crucial for understanding environmental changes, urbanization patterns, and resource management. Accurate LULC mapping relies on robust algorithms capable of interpreting complex datasets generated by modern remote sensing technologies. With the advent of high-resolution multiband satellite imagery, such as those provided by the Sentinel-2 mission, researchers have gained access to rich datasets that capture diverse spectral characteristics of the Earth’s surface, enabling enhanced LULC classification [37]. DL has emerged as a breakthrough approach in remote sensing, offering unparalleled performance in tasks such as object detection, segmentation, and classification [38,39]. By utilizing hierarchical feature extraction, DL models outperform traditional machine learning techniques in handling high-dimensional and heterogeneous satellite datasets. However, the development of effective DL models necessitates standardized benchmarks for comparative evaluation, reproducibility, and optimization [40]. The EuroSAT dataset, a labeled multiband satellite image dataset derived from Sentinel-2 data, has become a prominent benchmark in this field. It provides labeled samples across ten classes, such as urban, agricultural, and natural landscapes, representing diverse LULC categories. The dataset availability in multiple spectral bands, including red, green, blue (RGB), and near-infrared (NIR), facilitates the exploration of spectral and spatial features for LULC classification tasks [37]. As mentioned above, in this paper, we use the EuroSAT dataset based on Sentinel-2, which is widely used in the literature. A ranking of the performance of a large set of different methods using this dataset to validate their performance is available at https://paperswithcode.com/sota/image-classification-on-eurosat (accessed on 8 March 2025). The other tested satellite dataset is So2Sat LCZ42, which provides local climate zone (LCZ) labels for around 500,000 satellite image patches covering forty-two major urban areas and ten smaller regions worldwide. Labeled by fifteen domain experts over six months, it underwent a rigorous quality assessment. The dataset follows a 17-class LCZ classification scheme, distinguishing ten built and seven natural classes based on climate-relevant surface properties such as 3D structure, surface cover, and anthropogenic factors.

In this paper, we present two approaches for building an ensemble of neural networks. The first generates different three-channel images to fine-tune pretrained neural networks designed for RGB images. The second creates various multichannel images to train neural networks from scratch. We utilize three well-established architectures (ResNet50, MobileNetV2, and DenseNet201) pretrained on ImageNet, along with two custom architectures: one based on ResNet50 and the other on attention, both trained from scratch. The proposed ensemble is constructed by combining multiple networks using the sum rule, where each network is trained on a different three or multichannel image derived from the original multiband data. The proposed system achieves state-of-the-art (SOTA) performance across all datasets used in this study.

The contributions (both novelties and goals) in this paper are as follows:

- Presentation of a simple method for randomly creating three-channel images starting from multiband images, useful for CNNs pretrained on very large RGB image datasets, such as ImageNet;
- An example of EL based on DL architectures and the proposed approach for generating three or multichannel images from multiband images, where each network is trained on a given set of generated images (e.g., each network is trained on images created in the same way, where at least one channel is from one of the three RGB channels);
- Although computationally more expensive than other approaches, our proposed method is simple, consisting of only a few lines of code;
- We evaluate our approach using three datasets, following a clear and replicable protocol, thereby ensuring that our work can serve as a strong baseline for future research on multiband classification systems;
- Our approach obtains SOTA with no change in hyperparameters between datasets.
- The code of the proposed approach will be available at https://github.com/LorisNanni/Multi-Band-Image-Analysis-Using-Ensemble-Neural-Networks (accessed on 28 March 2025).

### Related Work

Mitra et al. [30] introduced a framework based on DL in 2019 that achieved moderate success over the performance of human experts (85% accuracy over 83%, respectively). Their approach, however, placed limited emphasis on the preprocessing stage, particularly the colorization process, potentially overlooking an opportunity to enhance performance further.

It is worth noting that the application of multigrayscale channel colorization to RGB is not confined to foraminifera classification; it extends to other fields as well. For example, in remote sensing [41,42], multispectral images often represented in grayscale can benefit from this technique, as can medical imaging, where grayscale formats like CT scans and MRI images [43] are prevalent. These colorization techniques can be employed on diverse classification challenges, including clinical diagnosis and image-guided interventions [44]. Studies have demonstrated that effective image fusion and the colorization of multiband representations can enhance the informational content of images [45]. This success indicates that improved preprocessing methods could enable DL frameworks to achieve superior performance, as shown in prior research [46]. The use of RGB colorization in these domains has already proven beneficial, improving classification accuracy and result interpretability. For instance, in medical imaging, converting grayscale images to RGB can make subtle features more discernible, potentially improving diagnostic accuracy [47]. Similarly, in remote sensing, RGB colorization can highlight specific elements, such as vegetation or water bodies, enhancing CNN performance [48]. Therefore, the proposed methods are likely to increase classification accuracy across various domains that rely on grayscale imagery. They may also perform well in image fusion tasks involving grayscale images from multispectral analyses [41,42] or polarized/filtered light sources used to capture objects outside the visible spectrum.

Additionally, ensemble learning, which combines different neural networks utilizing diverse image fusion techniques, has been shown to outperform individual methods significantly [49,50]. This result suggests that integrating multiple colorization approaches into ensembles could further boost the key performance indicators for the classification tasks under consideration.

## 2. Materials and Methods

This section provides an overview of both the datasets and neural networks utilized in this study. The foraminiferous dataset, originally introduced in [51], was analyzed using a convolutional neural network (CNN) ensemble. CNN ensembles are most effective when their individual models exhibit significant diversity [52,53]. In our approach, we employed well-established architectures, including ResNet50 (Res) [54], DenseNet201 (DN) [55], and MobileNetV2 (MV2) [56], all enhanced with transfer learning [57]. Numerous neural network-based methods have already been proposed for multi-band image classification [58,59,60,61].

### 2.1. Foramnifera Dataset

**Figure 1:**
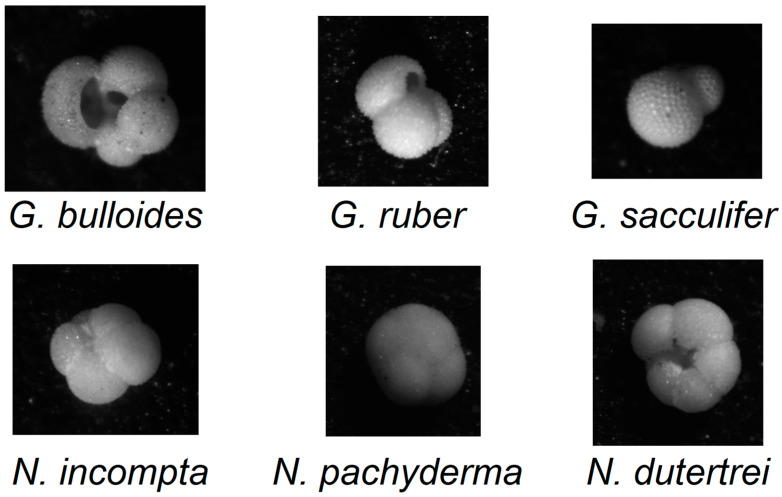
Visual examination of the six identified foraminifera species in the dataset.

The foraminifera dataset [51], see Figure 1 for the selected samples utilized in this study, is available at https://doi.pangaea.de/10.1594/PANGAEA.897873 (accessed on 17 December 2024).

It consists of 1437 samples categorized as follows:

- 178 images of *G. bulloides*;
- 182 images of *G. ruber*;
- 150 images of *G. sacculifer*;
- 174 images of *N. incompta*;
- 152 images of *N. pachyderma*;
- 151 images of *N. dutertrei*;
- 450 images labeled as “rest of the world”, representing other species of planktic foraminifera.

The original images were captured using a reflected light binocular microscope, with a light source positioned at 22.5° intervals. The equipment employed was an AmScope SE305R-PZ binocular microscope at 30× magnification [30]. For each foraminifera sample, sixteen grayscale images were taken under varying illumination angles. Image resolution varies across samples but is generally around 450 × 450 pixels. The testing protocol is four-fold cross-validation.

### 2.2. EuroSAT Dataset

Sentinel-2, a part of the European Union’s Earth observation program, Copernicus, provides high-resolution satellite images that are both openly accessible and freely available to the global research community. These satellite images capture detailed information across thirteen distinct spectral bands (see Table 1 for details), making them a valuable resource for diverse Earth observation applications.

**Figure 2:**
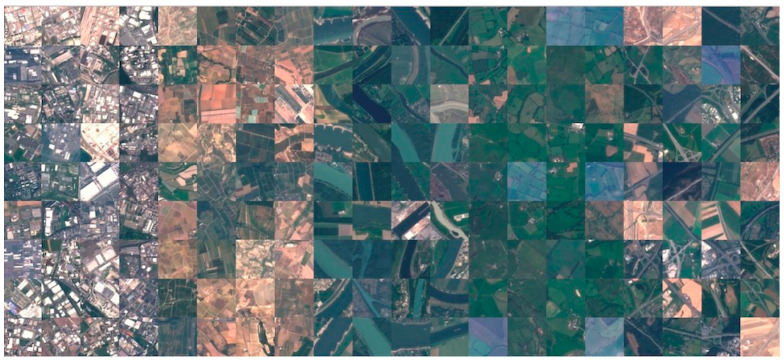
Visual comparison of some RGB images in the EuroSAT dataset.

This dataset [37] encompasses a comprehensive collection of 27,000 labeled images of size 64 × 64 pixels images. The images are georeferenced and meticulously categorized into ten distinct classes representing various land use and land cover types. Each class contains somewhere between 2000 and 3000 images. Samples are shown in Figure 2. The combination of multispectral data and precise geospatial referencing provides an unprecedented opportunity for researchers to develop and evaluate robust classification models. The ten classes are the following: industrial buildings, residential buildings, annual crop, permanent crop, river, sea and lake, herbaceous vegetation, highway, pasture, and forest.

For the testing protocol, we split the data into 80% for training and 20% for testing, as is standard practice. This dataset is available at https://github.com/phelber/eurosat (accessed on 17 December 2024). The official split into training/test sets is available at https://huggingface.co/datasets/torchgeo/eurosat/tree/main (accessed on 17 December 2024). The state-of-the-art (SOTA) results on this dataset are continuously updated at https://paperswithcode.com/sota/image-classification-on-eurosat (accessed on 8 March 2025).

It is important to note that the maximum value in each band is not 255, as in standard color images. For this reason, we normalize each band so that the maximum value becomes 255. The normalization parameters are extracted from the training set. Handling outliers is very simple: all values greater than one-tenth of the maximum value in the training set of a given band will take the value 255. Let *rec* be the maximum value in the training set of a given band. The normalization is conducted as follows:

rec = rec/10;

Image = Image./(rec/255);

Image = uint8(Image); %values are changed to 8-bit unsigned integers, so values greater than 255 become 255.

### 2.3. So2Sat LCZ42

The So2Sat LCZ42 dataset [62] comprises local climate zone (LCZ) labels for approximately half a million Sentinel-1 and Sentinel-2 image patches. These patches cover 42 major urban agglomerations and 10 smaller additional areas worldwide. The dataset was meticulously labeled by 15 domain experts over six months, following a carefully designed labeling workflow and evaluation process. Unlike many other labeled remote sensing datasets, So2Sat LCZ42 underwent a rigorous quality assessment by domain experts, achieving an overall confidence level of 85%. This dataset can be accessed from http://doi.org/10.14459/2018mp1483140 (last accessed on 8 March 2025).

**Figure 3:**
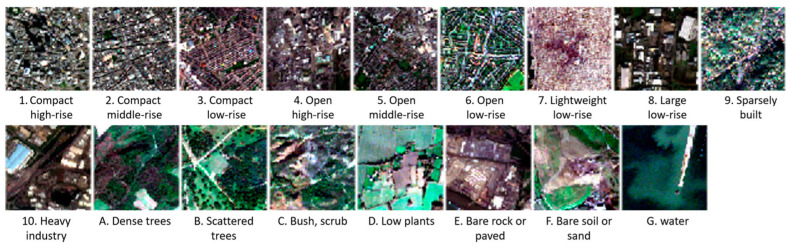
Visual comparison of some RGB images of the EuroSAT dataset.

The classification scheme used in this dataset consists of 17 classes: 10 built classes, and 7 natural classes. These classifications are based on climate-relevant surface properties at a local scale, including 3D surface structures (e.g., building and tree height and density), surface cover (e.g., vegetation or paving), and anthropogenic factors (e.g., human-induced heat emissions). Samples are shown in Figure 3. The selected 42 urban agglomerations and 10 smaller regions span all inhabited continents, excluding Antarctica.

The data in So2Sat LCZ42 contains the following 10 real-valued spectral bands:

1. Band B2—10 m GSD;
2. Band B3—10 m GSD;
3. Band B4—10 m GSD;
4. Band B5—upsampled to 10 m from 20 m GSD;
5. Band B6—upsampled to 10 m from 20 m GSD;
6. Band B7—upsampled to 10 m from 20 m GSD;
7. Band B8—10 m GSD;
8. Band B8a—upsampled to 10 m from 20 m GSD;
9. Band B11—upsampled to 10 m from 20 m GSD;
10. Band B12—upsampled to 10 m from 20 m GSD.

For machine learning applications, the dataset is divided into three subsets: training, testing, and validation sets, containing, respectively, 352,366, 24,188, and 24,119 image patch pairs (multispectral and synthetic aperture radar images). In this paper, we have used only multispectral images. The training set includes image patches from 32 cities plus the 10 additional smaller areas. The remaining 10 cities, distributed across various continents and cultural regions, are used for testing and validation. Specifically, each LCZ class label within these cities is split into western and eastern halves, forming the testing and validation sets. Thus, all three sub-datasets are geographically separated from each other despite having drawn the testing and validation sets from the same list of cities.

To ensure a realistic testing protocol, we did not use the validation set, as its images come from the same regions as the test set. Instead, our protocol treats the test set as an entirely independent dataset, ensuring that the cities used to extract test images are completely excluded from all stages of system development. This approach is commonly adopted in the literature to prevent data leakage and ensure a fair evaluation.

As we did with the EuroStat images, all were normalized to [0, 255], but more straightforwardly, since all channels are [0, 2.8], each image is normalized using Image=Image/(2.8/255).

### 2.4. CNN Ensemble Learning (EL)

**Figure 4:**
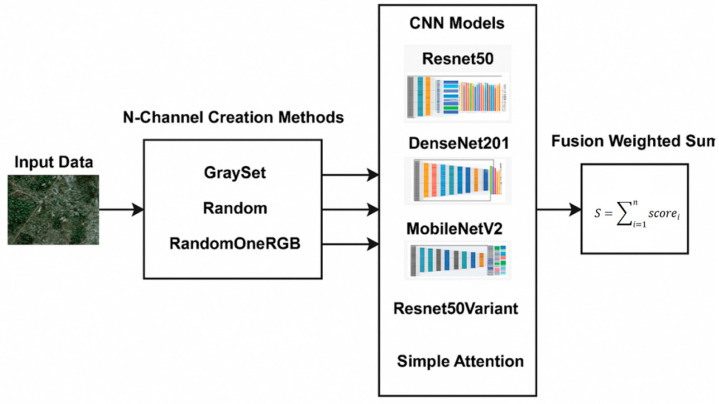
Schematic of our ensemble method.

The concept of EL is grounded in a straightforward principle: combining multiple models can lead to improved and more reliable results. Ensembles are most effective when the individual models exhibit significant diversity [52]. In this study, we construct ensembles by employing different techniques to represent input images as three-channel images or as multiband images. As illustrated in Figure 4, starting from the original multiband input image, we create different three-channel and multichannel images:

- The three-channel images are then used to train a set of three classic CNN networks, Resnet50, DenseNet201, and MobileNetV2, pretrained on ImageNet;
- The new multichannel images are then used to train a set of custom networks, one based on ResNet50 and the other on an attention model, both trained from scratch.

The predictions of the classifiers are combined using the sum rule, as shown in Figure 4.

#### 2.4.1. Ensemble Classifiers

Our ensemble is composed of CNNs and an attention-based network. CNNs were first introduced in the 1980s by French researcher Yann LeCun [53] for handwritten number classification and demonstrated strong performance throughout the 1990s [63,64]. Over the past decade, advancements in big data and GPU computing have significantly enhanced CNN performance, establishing them as the state-of-the-art approach in computer vision and image recognition. CNNs process three-dimensional tensors, often used to represent images of specific dimensions, where the width, height, and number of channels (e.g., RGB color values, alpha transparency, and depth) define the input.

In this work, we utilize three classic CNNs and two variants. The classic models are ResNet50 (Res) [54], DenseNet201 (DN) [55], and MobileNetV2 (MV2) [56], all pretrained on imageNet and available on MATLAB. ResNet-50 is a deep convolutional neural network with 50 layers that utilize residual (skip) connections to mitigate the vanishing gradient problem, allowing for efficient training of very deep networks. It consists of bottleneck residual blocks with batch normalization and ReLU activations, making it highly effective for image classification and feature extraction. DenseNet-201 is a 201-layer convolutional neural network that employs dense connectivity, where each layer receives inputs from all previous layers, promoting feature reuse and reducing the number of parameters. This architecture enhances gradient flow, mitigates redundancy, and improves efficiency in training deep networks. MobileNetV2 is a lightweight CNN designed for mobile and edge devices, utilizing depthwise separable convolutions and inverted residual blocks with linear bottlenecks to reduce computational complexity while maintaining high accuracy. Its efficient design makes it ideal for real-time applications with limited processing power. For these networks pretrained on ImageNet, we modify the head layer before initiating the fine-tuning process [65].

ResNet50, DenseNet201, and MobileNetV2 are fine-tuned further over 20 epochs (10 epochs for LCZ42 due to computational time), with a learning rate of 0.001 and a batch size of 30, using the SGD optimization approach. A key advantage of using pretrained networks is the application of transfer learning. This approach enables the model to leverage the knowledge acquired during prior training on one dataset and apply it to a new dataset [57]. This benefit is particularly valuable in DL models, which process vast arrays of weights and features. Transfer learning reduces both the training time and the quantity of data needed, making it ideal for relatively small datasets. In this study, all layers of the pretrained networks are fine-tuned, with none kept frozen.

In addition to ResNet50, DenseNet201, and MobileNetV2, we developed and tested a variant of ResNet50 and a custom network based on an attention model [66]. A generic attention model is a mechanism in neural networks that dynamically weighs input features based on their relevance to the task, allowing the model to focus on the most salient information while ignoring less relevant details. It computes attention scores by measuring the similarity between a query and a set of key-value pairs, typically using a dot-product or learned function. Scores are then normalized, often through a softmax function, to generate attention weights, which are applied to the values to produce a weighted sum as the output. This process enables the model to capture long-range dependencies and enhance performance in tasks such as machine translation, image captioning, and speech recognition. Both custom networks are implemented in PyTorch and optimized using the Adam optimizer.

##### ResNet-Based Custom Architectures (Cres)

Our network leverages the strengths of both GoogLeNet’s Inception Block and ResNet’s Residual Block by integrating them into a unified architecture. It consists of three sequential macroblocks, each built by embedding an Inception Block within a Residual Block. We acknowledge that several papers have already explored combining GoogleNet and ResNet. However, our goal is not to build our network so that it competes with the literature but rather to introduce an alternative architecture that differs from the conventional CNNs used in our ensemble.

Initially, a multichannel input image undergoes a convolution with 64 filters, followed by 2 × 2 max pooling. After the final macroblock, the output of size 512 × 4 × 4 is flattened and passed through a feed-forward network composed of three fully connected layers, ultimately producing logits matching the size of the number of classes.

Within each macroblock, the Inception Block replicates the branch structure detailed in the original paper by employing different combinations of small convolutional filters to extract diverse features. With four branches, each convolutional layer in a branch uses one-fourth of the filters allocated for the final Inception output. The branch outputs are concatenated and then combined with a 1 × 1 convolution of the Residual Block’s input to ensure matching dimensions. The 1 × 1 convolution is crucial for aligning the original input with the new output shape. While the number of channels doubles at each stage, the spatial dimensions remain constant within each block; 2 × 2 max pooling is applied after each block to progressively reduce height and width. All convolutional and dense layers use the ReLU activation function, and every convolutional layer (except for the 1 × 1 convolution in the Residual Block) is followed by a Batch Normalization layer.

**Figure 5:**
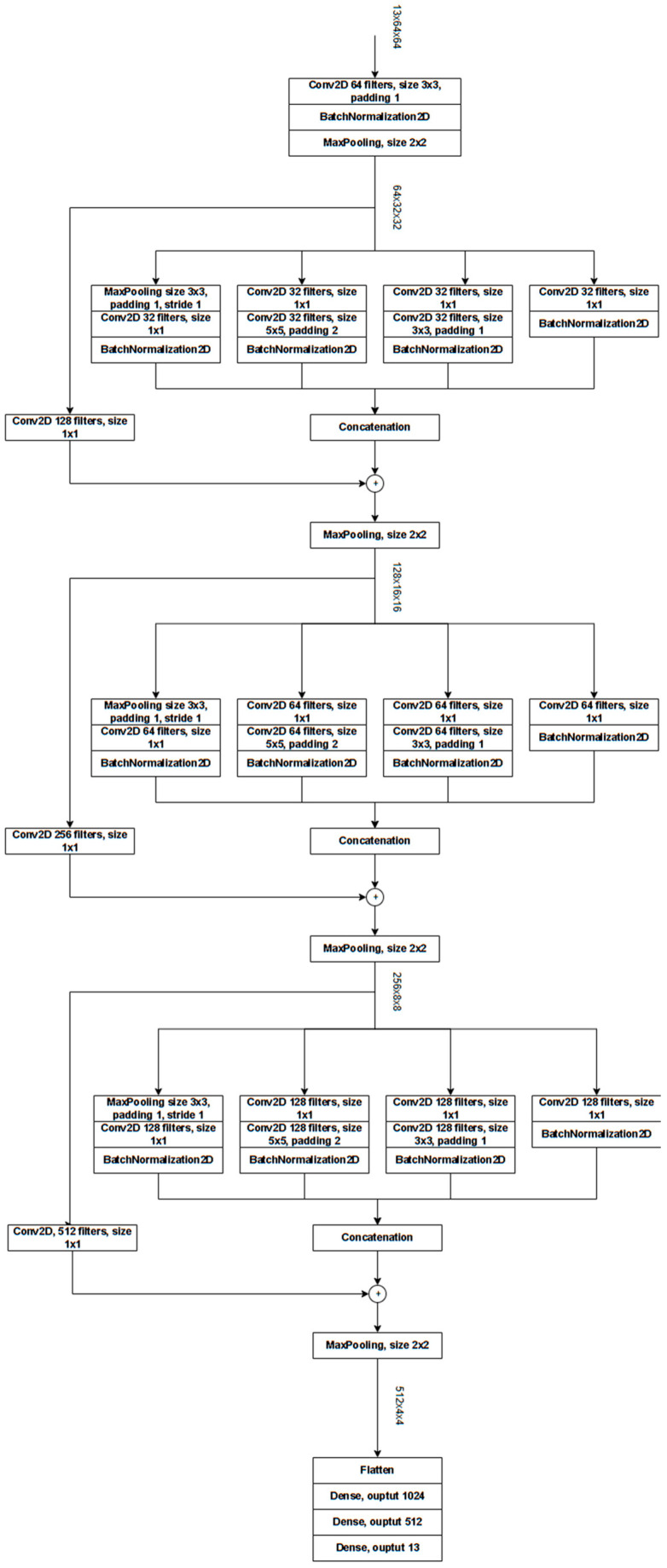
Schematic of Cres: in this example, the input is a 13-channel image (EuroSat).

The architecture is detailed in Figure 5. We use Adam optimization with an initial learning rate of 1 × 10^−3^, and we apply a step scheduler with a step size of 5 and a decay factor (gamma) of 0.5. The network is trained using cross-entropy loss. Before the sum rule, the scores of this network are normalized using softmax.

##### Custom Attention-Based Network (Catt)

**Figure 6:**
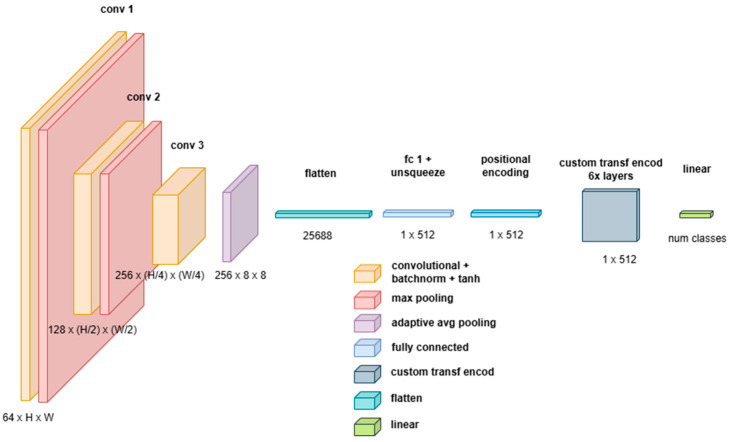
Schematic of Catt.

This network combines the local feature extraction capabilities of 3D Convolutional Neural Networks (CNNs) with the long-range dependency modeling of Transformer-based architectures, see Figure 6. The proposed architecture effectively extracts multiscale spatial and spectral representations via a 3D CNN backbone before processing them through a Transformer Encoder, which refines feature dependencies and enhances global context modeling. The architectural design ensures computational efficiency and robustness in handling high-dimensional input data.

The model Is composed of the following two key components:

1. A 3D CNN Backbone for hierarchical feature extraction and dimensionality reduction;
2. A Transformer Encoder for long-range dependency modeling and final classification.

The 3D CNN backbone consists of three convolutional blocks, each composed of the following:

1. A 3D convolutional layer (Conv3D) with a kernel size of 3×3×3, enabling local feature extraction across spatial and spectral dimensions;
2. Batch normalization (BatchNorm3D) to normalize activations and improve stability during training;
3. A non-linearity (Tanh activation function), which normalizes values in the range $\left[-1,1\right]$.

The initial processing of the input occurs through a stack of 3D convolutional layers, defined as follows:

$$x^{\prime }=\sigma \left(BatchNorm(Conv3D(x))\right)$$

where x is the input tensor, σ is the Tanh activation function, and batch normalization ensures stability. This is followed by a 3D Max Pooling operation, as follows:

$$x_{pool}=MaxPool3D(x^{\prime })$$

Each convolutional block extracts increasingly abstract feature representations, refining the learned feature space. Notably, the pooling operations are carefully designed to prevent the depth dimension from being reduced excessively. Specifically:

- The first two pooling layers use a kernel of 1×2×2, reducing only the height and width but keeping the spectral depth unchanged;
- The final layer employs Adaptive Average Pooling, ensuring a fixed-size feature representation of 1×8×8, regardless of input image size.

The final convolutional feature map is then flattened and projected into an embedding space suitable for Transformer processing.

The adoption of a 3D CNN module provides several key advantages over traditional 2D CNN architectures, as follows:

1. Preserves multispectral and volumetric information; unlike 2D CNNs, which process each spectral channel independently or in stacked formats, 3D CNNs treat spectral and spatial dimensions jointly, maintaining inter-band relationships;
2. Reduces computational complexity for the Transformer; the 3D CNN acts as a preprocessing mechanism, reducing the input dimensions passed to the Transformer, thereby helping to mitigate the computational overhead of self-attention mechanisms, which scales quadratically with sequence length;
3. Results in robust Spatial Feature Extraction: CNNs provide strong inductive biases for grid-structured data, capturing local correlations in a way that self-attention mechanisms alone struggle to replicate;
4. Provide adaptive representation learning; the use of adaptive average pooling guarantees that the Transformer receives a consistent input size, preventing issues with variable input resolutions.

After feature extraction via the 3D CNN, the resulting feature vectors are fed into a Transformer Encoder, which refines global dependencies and long-range interactions within the extracted representations.

The Transformer module consists of six stacked Transformer Encoder Layers, each performing Multi-Head Self-Attention (MHSA) and Feed-forward Processing. Since Transformers do not inherently understand spatial relationships, we incorporate positional encodings in a standard way, encoding position information using sine and cosine functions, as follows:

$$PE_{\left(pos,2i\right)}=sin\left(\frac{pos}{10000^{\frac{2i}{d_{model}}}}\right),$$

$$PE_{\left(pos,2i+1\right)}=cos\left(\frac{pos}{10000^{\frac{2i}{d_{model}}}}\right).$$

The MHSA module allows the model to capture global dependencies across the feature space, as follows:

$$Attention(Q,K,V)=Softmax\left(\frac{QK^{T}}{\sqrt{d_{k}}}\right)V$$

where Q,K,V are linear projections of the input embeddings, and oftmax normalization ensures that attention weights sum to 1.

Each attention head operates independently, and its outputs are concatenated and projected into the original embedding space.

Each Transformer layer contains a position-wise feed-forward network (FFN):

$$FFN(X)=max\left(0,XW_{1}+b_{1}\right)W_{2}+b_{2}$$

Additionally, Layer Normalization and residual connections stabilize training.

#### 2.4.2. Fusion Method

The fusion method employed is the sum rule [50], defined as follows:

$$sum=\sum_{i=1}^{N}v;out=argmax\left\{{sum}_{j}\right\},j=1\ldots n$$

Here, *N* represents the number of models, and *n* denotes the size of each confidence vector **v**.

The sum rule is considered one of the most effective fusion methods, as it avoids potentially harmful operations such as multiplication by zero.

### 2.5. Three/Multichannel Image Creation

The proposed ensemble is constructed by combining multiple networks using the sum rule, where each network is trained on a different three or multichannel image derived from the original multiband data.

#### 2.5.1. Three-Channel Image Creation

Since we utilize pretrained networks that require RGB images as the input, the initial step in our pipeline involves converting the dataset into CNN-compatible inputs.

The GraySet method achieves this by transforming each of the sixteen grayscale images within a pattern into RGB images. This transformation is accomplished by replicating the grayscale values across all three color channels, resulting in sixteen RGB images for each training and testing pattern. Specifically, for a given pattern comprising sixteen grayscale images (denoted as $I_{1}$ … $I_{16}$), it is encoded into an RGB image following this rule:

1. $R\left(x\right)=I_{1},\;G\left(x\right)=I_{1},\;B\left(x\right)=I_{1}$ becomes the first RGB image;
2. $R\left(x\right)=I_{2},\;G\left(x\right)=I_{2},\;B\left(x\right)=I_{2}$ becomes the second RGB image;
3. $R\left(x\right)=I_{16},\;G\left(x\right)=I_{16},\;B\left(x\right)=I_{16}$ becomes the last RGB image.

This transformation is applied consistently across both training and testing patterns. For each test pattern, the method generates sixteen RGB images, leading to sixteen scores produced by the trained CNN. These scores are then combined using the average rule to produce the final result.

In the Random method, the three channels are obtained simply by randomly extracting three channels from the channels of the multiband images.

In the RandomOneRGB method, applied to the EuroSat and LCZ42 datasets, the performance of the RGB channels is higher than the other channels, so the following procedure is used: two channels are randomly drawn among the thirteen bands, and the other channel is randomly drawn among the three *R*, *G*, and *B* bands.

Since we are generating images to work well with an ensemble, there are no constraints imposed on the random draw. Thus, it is possible for a network in our ensemble to be trained on multiband images transformed, for instance, into *RRR*, i.e., on three red channels in RGB.

#### 2.5.2. Multichannel Image Creation

In the case of the Foramnifera dataset, we extract 16 channels from the original set using random sampling with replacement, essentially adopting a bagging approach for ensemble creation. Consequently, the same channel can appear multiple times in the newly created multichannel images. Each new set of images is then used to train a distinct neural network.

For the EuroSat and LCZ42 datasets, we apply a similar strategy as in Foramnifera (with EuroSat having 13 channels and LCZ42 10 channels). Recognizing that the RGB channels consistently deliver higher performance, we adopt the following procedure: the first channel of each new multichannel image is randomly selected from the R, G, and B bands. As described in Section 2.5.1, our image generation process is designed to optimize ensemble performance, so no additional constraints are imposed on the random selection.

**Figure 7:**
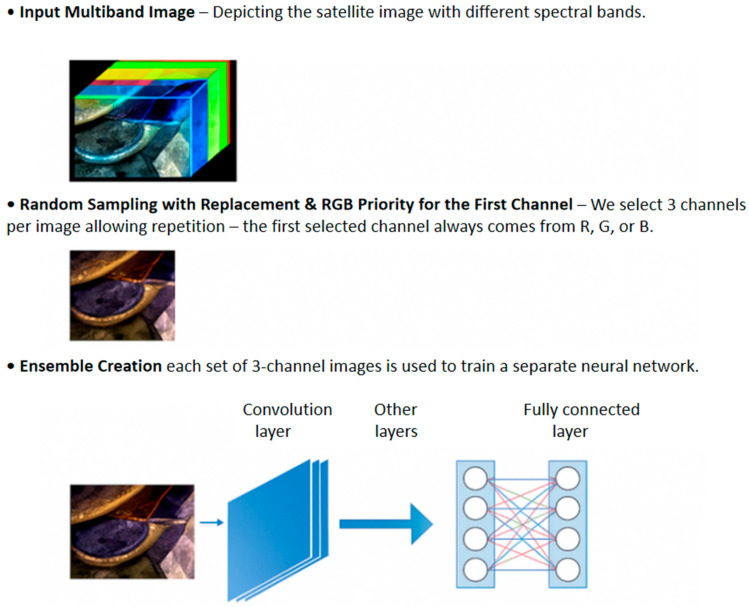
Multichannel image creation for feeding pretrained CNN. Different colored lines represent shared weights.

In Figure 7, we show how multichannel image creation works for feeding pretrained CNN.

### 2.6. Performance Metrics

Precision, F1-score, and accuracy are the evaluation metrics for evaluating the classification models in this study. Precision measures the proportion of correctly predicted positive instances among all predicted positives, F1-score balances precision and recall as their harmonic mean, and accuracy represents the proportion of correctly classified instances over the total number of instances. Given the number of True Positives (TPs), False Positives (FPs), True Negatives (TNs), and False Negatives (FNs), each of these metrics can be defined as follows:

$$\mathrm{Precision}=\frac{TP}{TP+FP}$$

$$\mathrm{Recall}=\frac{TP}{TP+FN}$$

$$F1-\mathrm{score}=\frac{2(Precision*Recall)}{Precision+Recall}=\frac{2TP}{2TP+FP+FN}$$

$$\mathrm{Accuracy}=\frac{TP+TN}{TP+TN+FP+FN}$$

## 3. Results

For the Foramnifera and EuroSat datasets, the applied data augmentation takes a given image and randomly reflects it top–bottom and left–right to produce two new images. The third transform linearly scales the original image along both axes with two factors randomly extracted from the uniform distribution [1,2]. For the LCZ42 dataset, we did not apply any data augmentation techniques, as the original dataset size is sufficiently large.

In Table 2, the results on the Foramnifera dataset are reported. We compare the F1-scores reported in the literature with our proposed approaches. The value between the parentheses ’()’ of each approach is the number of combined networks, e.g., Random(10) means that we trained ten networks using the method Random detailed in Section 2.4, and then these networks were combined by the sum rule. The testing protocol for the dataset is the four-fold cross-validation, and the performance metric is the F-score as in the related literature.

In Table 2, the following applies:

- Y(*t*)_X means that we coupled the X architecture with ensemble Y, where the ensemble has *t* networks;
- X + Z means that we combine by sum rule the X and Z architectures with networks coupled with Random(20);
- X + Y + Z_z means that we combine by sum rule the X, Y, and Z architectures with networks coupled with Random(z).

In Table 3, the precision, recall, accuracy, and $F_{1}$ of different methods are compared.

The results in Table 2 and Table 3 clearly show that many ensembles proposed in this work exceed the current SOTA in the foraminifera dataset; even the tiny set of six networks (Res+DN+MV2_2) gets a higher F1 than the previous SOTA. The two custom scratch-trained networks achieve very low performance. The size of the training set is not sufficient to train a network from scratch. Catt does a little better in Res+DN+MV2. It improves the baseline (Res+DN+MV2+Catt improves Res+DN+MV2), but its behavior is not stable. Finally, DN+MV2 is shown to outperform DN+MV2+Catt.

The following approaches are reported in Table 4 for the EuroSAT satellite data:

- RGB(x) means that x networks are trained using RGB channels and then combined with the sum rule;
- X + Z means that we combine by sum rule the X and Z architectures, both coupled with RandomOneRGB(20);
- X + Y + Z_1 means that we combine by sum rule the X, Y, and Z architectures, coupled with RandomOneRGB(1).

As noted, the performance accuracy of many approaches is reported in https://paperswithcode.com/sota/image-classification-on-eurosat (accessed on 8 March 2025). Here, we report only the four best that did not use extra training data.

Various vision transformer models evaluated on EuroSat are discussed in [66]. The study aims to identify the most effective pretrained model for land use classification in onboard satellite processing by prioritizing high accuracy, computational efficiency, and resilience to noisy data. Among the assessed approaches, Apple’s MobileViTV2 delivered the best performance.

The results shown In Table 4 show that our system achieves performance similar to that of the current SOTA.

**Table 4:** The accuracy obtained by each methodology on the EuroSAT dataset.

Approach	Accuracy
RGB(1)_Res	98.54
RGB(5)_Res	98.80
Random(5)_Res	98.57
RandomOneRGB(5)_Res	98.91
RandomOneRGB(20)_Res	98.94
RandomOneRGB(20)_DN	99.15
RandomOneRGB(20)_MV2	99.22
RandomOneRGB(20)_Cres	98.87
RandomOneRGB(20)_Catt	98.24
Res+DN	99.17
Res+DN+MV2	99.22
DN+MV2	99.20
Res+DN+MV2+Catt	99.39
Res+DN+MV2+Cres	**99.41**
Res+DN+MV2+Catt+Cres	99.33
Res+DN+MV2_1	99.02
Res+DN+MV2+Catt _1	99.20
Res+DN+MV2+Cres_1	99.28
Res+DN+MV2+Catt+Cres_1	99.22
[58]	99.24
[59]	99.22
[60]	99.20
[61]	98.96
MobileViTV2 (Apple) [66]	99.09
ViT-large (Google) [66]	98.55
SwinTransformer (Microsoft) [66]	98.83

The accuracy reported in Table 4 shows that the ensemble, based on the ResNet50 network, RandomOneRGB() outperforms the ensemble Random() and that the ensemble Random() outperforms the ensemble RGB().

Some ensembles proposed here outperform SOTA; the small ensemble of just four networks (Res+DN+MV2+Cres_1) achieves results lower than our best ensemble, but it still surpasses the current state of the art.

Finally, in Table 5, we report performance on the LCZ42 dataset. Due to computational constraints, only a subset of the tests conducted for EuroSAT was run for the LCZ42 dataset. Since DenseNet is computationally expensive to tune, we trained only three networks. Despite this, our DenseNet ensemble achieved performance comparable to ensembles of the 10 MobileNet and 10 ResNet models. To ensure a balanced contribution from all architectures in the final ensemble using the sum rule, each DenseNet model was assigned a weight of 3. This adjustment compensates for the smaller number of DenseNet networks (3 vs. 10 for others), effectively making the sum rule a weighted sum rule.

Notice that [67] proposed a prior knowledge coupling (PKC) module. This module was created by evaluating system performance on the validation set, which, importantly, is drawn from the same cities as the test set. Therefore, using this module leads to an unfair comparison with our method, as we do not utilize any information from the validation set. The test set in our approach is a genuinely independent set of cities. In [68], the authors report the highest accuracy on LCZ42 (74.42%), but both multispectral and synthetic aperture radar images were used. Consequently, our performance is not comparable because [68] uses multiple sources of information.

Apart from the papers mentioned above, in all cases where the comparison between our ensemble and the literature is entirely fair, our proposed ensemble achieves SOTA for the LCZ42 dataset as well.

**Table 5:** The accuracy obtained by each methodology on the LCZ dataset. * Considering the methods reported in [67], our method is fairly comparable only with “without PKC”, as above explained.

Approach	Year	Accuracy
RandomOneRGB(1)_Res	2025	68.67
RandomOneRGB(1)_MV2	2025	64.78
RandomOneRGB(1)_DN	2025	66.72
RandomOneRGB(1)_Cres	2025	62.54
RandomOneRGB(10)_Res	2025	70.97
RandomOneRGB(10)_MV2	2025	70.91
RandomOneRGB(3)_DN	2025	70.96
RandomOneRGB(10)_Cres	2025	67.21
Res+MV2	2025	71.80
Res+MV2+DN	2025	72.42
Res+MV2+Cres	2025	72.37
Res+MV2+Cres+DN	2025	**72.79**
[62]	2020	61.10
[69]	2023	67.87
[70]	2023	68.51
[71]	2020	69.40
[72]	2023	70.00
[67] without PKC *	2024	71.10
[67] with PKC *	2024	73.80

## 4. Discussions

It is interesting to note that our ensembles are simple to create, and their performance is comparable, or better, with current SOTA. The primary drawback of the proposed ensemble method is the increased computational time. Nevertheless, using a NVIDIA, Santa Clara, CA, USA, Titan RTX 24 GB GPU from 2018 with 4608 CUDA cores (for comparison, the current NVIDIA, Santa Clara, CA, USA, 5090 has 21,760 CUDA cores), a batch of 10,000 images can be classified by pretrained networks as follows (model, time):

- ResNet50, 10.86 s;
- DenseNet201, 97.19 s;
- MobileNetV2, 9.42 s.

Such times are certainly not a problem in applications where high computing power servers can be used to perform the classification of all the images. Since the networks are combined using the simple sum rule, we can parallelize the process across multiple GPUs if available. Regardless, the proposed ensembles are clearly not suited for on-device approaches.

Several strategies can be employed to reduce the computational burden while maintaining performance with CNNs [73,74]. One method is knowledge distillation [74], a widely used approach for compressing an ensemble into a single, smaller model. The ensemble’s knowledge is transferred to a more efficient student network by training it to mimic the predictions of the ensemble, typically using a softened softmax output. This method significantly reduces inference time while retaining much of the ensemble’s accuracy.

Another method is quantization [75]. It reduces the precision of weights and activations from 32-bit floating-point representations to lower-bit formats such as 16-bit or 8-bit integers. This process can lead to significant speedup with minimal accuracy degradation, especially when supported by hardware accelerations such as Tensor Processing Units (TPUs) and Graphics Processing Units (GPUs).

Instead of evaluating all models in the ensemble, a dynamic subset of models can be selected for inference based on input characteristics. Techniques like gating networks or adaptive inference allow for selective model execution, reducing computation without significantly impacting accuracy.

Excluding applications with limited computational power—such as embedded systems or scenarios lacking server access (e.g., on-device satellite classification)—the ensemble approach clearly yields SOTA performance. Although it demands more computational resources than some alternatives, the ensemble is straightforward to implement, taking advantage of all available networks, whether pretrained or our custom available models. Moreover, it is applicable across a wide range of datasets where patterns are represented as multiband images.

Finally, we conducted a hyperparameter sensitivity analysis by varying key training parameters such as the learning rate, batch size, and number of epochs. The results are reported in Table 6. This analysis shows that CNNs are robust to training parameters. For the sake of computation time, we ran experiments using Res+DN+MV2_1 in the Foramnifera dataset.

The training parameters were not tailored specifically to any one dataset; instead, we employed a set of standard hyperparameters consistently across all three datasets examined in this study (to avoid any overfitting). The results clearly demonstrate that the network performance remains stable within a defined range of hyperparameter values.

**Figure 8:**
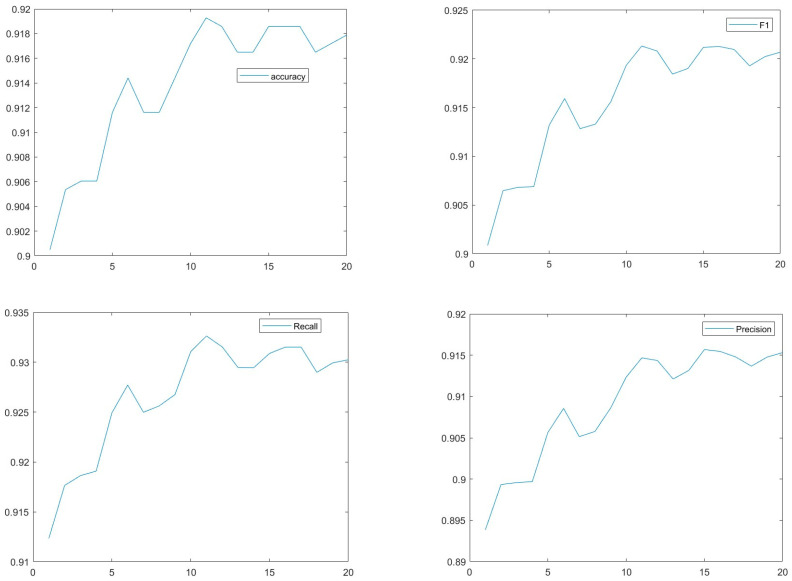
Res+DN+MV2 model performance (y-axis) vs. size of the ensemble (x-axis). The Foraminifera dataset was used in this test.

Beyond loss and accuracy, we added plots (size ensemble vs. Metric), see Figure 8, for other important metrics (e.g., precision, recall, and F1-score) to give a comprehensive view of the ensemble Res+DN+MV2 model performance (y-axis) vs. Size of the ensemble (x-axis). The Foraminifera dataset was used in this test. Performance improves dramatically when the ensemble size increases from 1 to 10; however, further increases from 10 to 20 yield only modest improvement.

## 5. Conclusions

Although the experiments presented in this paper are focused on only three datasets (representing two very different problems), the impact of ensemble DL is clearly evident. Although classification using multiple neural nets proved to be time intensive, our efforts directed toward optimizing accuracy were met with considerable success. As we show, our ensembling method for multiband images significantly improves classification performance compared to SOTA—such a level of performance positions the model as a valuable tool for the tasks at hand. The substantial improvement in key performance indicators, with respect to stand-alone networks, justifies the computational trade-offs, highlighting the effectiveness of a robust ensemble method over single neural networks for image classification tasks.

Our work fills significant gaps in the field through the following:

- Bridging model architectures: we combine the strengths of standard CNNs and custom architectures through an ensemble approach, which not only achieves state-of-the-art performance but also offers a more accessible alternative compared to methods that rely solely on highly complex or custom networks;
- Providing ease and availability of implementation: all source code used in this study is freely available on GitHub. In this way, we enhance reproducibility and lower the barrier for researchers and practitioners. This ease of implementation addresses the gap where many SOTA methods are difficult to deploy or replicate.

To summarize, our contribution not only advances the technical state-of-the-art in multichannel image classification but also makes a significant impact by being easy to implement and more readily deployable in practical, cloud-based settings.

In future work, we plan on building on the promising results presented in this study through the following:

- Expanding model architectures: while our ensemble incorporates established CNN models and custom architectures, exploring additional deep learning frameworks—such as more transformer-based models or graph neural networks—could further capture the complex spectral and spatial dependencies inherent in multiband data. Thus, we plan on developing more custom networks for managing both multispectral and synthetic aperture radar images;
- Investigating domain adaptation and transfer learning: future work could focus on leveraging domain adaptation strategies to enable the ensemble framework to generalize across different sensor types and imaging conditions. Incorporating transfer learning could also facilitate rapid adaptation to new datasets, particularly in dynamic environmental monitoring scenarios;
- Providing temporal dynamics and multitemporal analysis: extending the current framework to handle time-series multiband images will allow for the analysis of temporal changes in land cover or environmental conditions. This integration would enable monitoring of evolving phenomena and improve the relevance of the approach for real-time applications.
- Exploring scalability and real-time implementation: exploring the scalability of the proposed system in operational environments is essential. Optimizing the framework for real-time processing and deployment—possibly through distributed computing or edge-based implementations—could significantly expand its practical utility in remote sensing applications.

These directions aim to address current limitations and pave the way for more robust and adaptable models in the challenging domain of multiband image classification.

## Figures and Tables

**Table 1:** All thirteen bands captured by Sentinel-2’s Multispectral Imager (MSI) are detailed, including their identification, spatial resolution, and central wavelength for each spectral band.

Band	Spatial Central Resolution—Meters	Wavelength—Nanometer
B01—Aerosols	60	443
B02—Blue	10	490
B03—Green	10	560
B04—Red	10	665
B05—Red edge 1	20	705
B06—Red edge 2	20	740
B07—Red edge 3	20	783
B08—NIR	10	842
B08A—Red edge 4	20	865
B09—Water vapor	60	945
B10—Cirrus	60	1375
B11—SWIR 1	20	1610
B12—SWIR 2	20	2190

**Table 2:** Results obtained with each methodology on the Foraminifera dataset.

Approach	F1-Measure
[30]	85.0
[35]	90.6
GraySet(10)_Res	89.4
Random(10)_Res	91.1
Random(20)_Res	91.3
Random(20)_DN	91.5
Random(20)_MV2	90.2
Random(10)_Cres	62.7
Random(20)_Catt	69.7
Res+DN	91.8
Res+DN+MV2	92.1
DN+MV2	92.3
Res+DN+MV2+Catt	**92.5**
DN+MV2+Catt	92.1
Res+DN+MV2_1	90.1
Res+DN+MV2_2	90.7
Res+DN+MV2_3	90.7

**Table 3:** Comparison (precision, recall, accuracy, and $F_{1}$ score) between the previous SOTA and the best ensemble presented here.

	Precision (%)	Recall (%)	*F*_1_ Score (%)	Accuracy (%)
Human Novices (max) [30]	65	64	63	63
Human Experts (max) [30]	83	83	83	83
ResNet50 + Vgg16 [30]	84	86	85	85
Stand alone Vgg16 [30]	80	82	81	81
[35]	90.9	90.6	90.6	90.7
Res+DN	91.1	92.8	91.8	91.7
Res+DN+MV2	91.5	93.0	92.1	91.8
DN+MV2	91.6	93.4	92.3	92.0
Res+DN+MV2+Catt	**91.9**	**93.5**	**92.5**	**92.2**

**Table 6:** Hyperparameter sensitivity analysis.

Res+DN+MV2_1	Batch Size = 20	Batch Size = 30	Batch Size = 60
Epochs = 15	89.9	90.0	90.1
Epochs = 20	90.1	90.1	90.2
Epochs = 30	90.1	90.0	90.0

## Data Availability

Foramnifera: https://doi.pangaea.de/10.1594/PANGAEA.897873 (accessed on 28 March 2025); EuroSAT: https://zenodo.org/records/7711810 (accessed on 28 March 2025); LCZ42: https://dataserv.ub.tum.de/index.php/s/m1483140 (accessed on 28 March 2025).

## References

[1] Nalepa J. (2021). Recent Advances in Multi- and Hyperspectral Image Analysis. Sensors.

[2] Uddin M.P., Mamun M.A., Hossain M.A. Feature extraction for hyperspectral image classification. Proceedings of the 2017 IEEE Region 10 Humanitarian Technology Conference (R10-HTC).

[3] Chen H., Miao F., Chen Y., Xiong Y., Chen T. (2021). A hyperspectral image classification method using multifeature vectors and optimized KELM. IEEE J. Sel. Top. Appl. Earth Obs. Remote Sens..

[4] Shi G., Huang H., Wang L. (2019). Unsupervised dimensionality reduction for hyperspectral imagery via local geometric structure feature learning. IEEE Geosci. Remote Sens. Lett..

[5] Xu X., Li J., Li S., Plaza A. (2019). Subpixel component analysis for hyperspectral image classification. IEEE Trans. Geosci. Remote Sens..

[6] Zhang X., Jiang X., Jiang J., Zhang Y., Liu X., Cai Z. (2021). Spectral–spatial and superpixelwise PCA for unsupervised feature extraction of hyperspectral imagery. IEEE Trans. Geosci. Remote Sens..

[7] Champa A.I., Rabbi M.F., Hasan S.M.M., Zaman A., Kabir M.H. Tree-based classifier for hyperspectral image classification via hybrid technique of feature reduction. Proceedings of the 2021 International Conference on Information and Communication Technology for Sustainable Development (ICICT4SD).

[8] Wei L., Huang C., Wang Z., Wang Z., Zhou X., Cao L. (2019). Monitoring of urban black-odor water based on Nemerow index and gradient boosting decision tree regression using UAV-borne hyperspectral imagery. Remote Sens..

[9] Xu S., Liu S., Wang H., Chen W., Zhang F., Xiao Z. (2020). A hyperspectral image classification approach based on feature fusion and multi-layered gradient boosting decision trees. Entropy.

[10] Bazine R., Huayi W., Boukhechba K. K-NN similarity measure based on fourier descriptors for hyperspectral images classification. Proceedings of the 2019 International Conference on Video, Signal and Image Processing.

[11] Bhavatarini N., Akash B.N., Avinash A.R., Akshay H.M. Object detection and classification of hyperspectral images using K-NN. Proceedings of the 2023 Second International Conference on Electrical, Electronics, Information and Communication Technologies (ICEEICT).

[12] Zhang L., Huang D., Chen X., Zhu L., Xie Z., Chen X., Cui G., Zhou Y., Huang G., Shi W. (2023). Discrimination between normal and necrotic small intestinal tissue using hyperspectral imaging and unsupervised classification. J. Biophotonics.

[13] Chen G.Y. (2021). Multiscale filter-based hyperspectral image classification with PCA and SVM. J. Electr. Eng..

[14] Zhang S., Huang H., Huang Y., Cheng D., Huang J. (2022). A GA and SVM classification model for pine wilt disease detection using UAV-based hyperspectral imagery. Appl. Sci..

[15] Pathak D.K., Kalita S.K., Bhattacharya D.K. (2022). Hyperspectral image classification using support vector machine: A spectral spatial feature based approach. Evol. Intell..

[16] Jain V., Phophalia A. Exponential weighted random forest for hyperspectral image classification. Proceedings of the IGARSS 2019-2019 IEEE International Geoscience and Remote Sensing Symposium.

[17] Kishore K.M.S., Behera M.K., Chakravarty S., Dash S. Hyperspectral image classification using minimum noise fraction and random forest. Proceedings of the 2020 IEEE International Women in Engineering (WIE) Conference on Electrical and Computer Engineering (WIECON-ECE).

[18] Zhao J., Yan H., Huang L. (2023). A joint method of spatial–spectral features and BP neural network for hyperspectral image classification. Egypt. J. Remote Sens. Space Sci..

[19] Li Z., Liu F., Yang W., Peng S., Zhou J. (2022). A Survey of Convolutional Neural Networks: Analysis, Applications, and Prospects. IEEE Trans. Neural Netw. Learn. Syst..

[20] Khan A., Rauf Z., Sohail A., Khan A.R., Asif H., Asif A., Farooq U. (2023). A survey of the vision transformers and their CNN-transformer based variants. Artif. Intell. Rev..

[21] Liu S., Chu R.S.W., Wang X., Luk W. (2019). Optimizing CNN-based hyperspectral image classification on FPGAs. International Symposium on Applied Reconfigurable Computing.

[22] Butt M.H.F., Ayaz H., Ahmad M., Li J.P., Kuleev R. A fast and compact hybrid CNN for hyperspectral imaging-based bloodstain classification. Proceedings of the 2022 IEEE Congress on Evolutionary Computation (CEC).

[23] Bhosle K., Ahirwadkar B. (2021). Deep learning convolutional neural network (cnn) for cotton, mulberry and sugarcane classification using hyperspectral remote sensing data. J. Integr. Sci. Technol..

[24] Yan T., Xu W., Lin J., Duan L., Gao P., Zhang C., Lv X. (2021). Combining multi-dimensional convolutional neural network (CNN) with visualization method for detection of aphis gossypii glover infection in cotton leaves using hyperspectral imaging. Front. Plant Sci..

[25] Yu C., Han R., Song M., Liu C., Chang C.-I. (2020). A simplified 2D-3D CNN architecture for hyperspectral image classification based on spatial–spectral fusion. IEEE J. Sel. Top. Appl. Earth Obs. Remote Sens..

[26] Ghaderizadeh S., Abbasi-Moghadam D., Sharifi A., Zhao N., Tariq A. (2021). Hyperspectral image classification using a hybrid 3D-2D convolutional neural networks. IEEE J. Sel. Top. Appl. Earth Obs. Remote Sens..

[27] Xu Q., Yuan X., Ouyang C., Zeng Y. (2020). Attention-based pyramid network for segmentation and classification of high-resolution and hyperspectral remote sensing images. Remote Sens..

[28] Mdrafi R., Du Q., Gurbuz A.C., Tang B., Ma L., Younan N.H. (2020). Attention-based domain adaptation using residual network for hyperspectral image classification. IEEE J. Sel. Top. Appl. Earth Obs. Remote Sens..

[29] Zhang M., Gong M., He H., Zhu S. (2020). Symmetric all convolutional neural-network-based unsupervised feature extraction for hyperspectral images classification. IEEE Trans. Cybern..

[30] Mitra R., Marchitto T.M., Ge Q., Zhong B., Kanakiya B., Cook M.S., Fehrenbacher J.S., Ortiz J.D., Tripati A., Lobaton E. (2019). Automated species-level identification of planktic foraminifera using convolutional neural networks, with comparison to human performance. Mar. Micropaleontol..

[31] Edwards R., Wright A. (2015). Foraminifera. Handbook of Sea-Level Research.

[32] Liu S., Thonnat M., Berthod M. Automatic classification of planktonic foraminifera by a knowledge-based system. Proceedings of the Tenth Conference on Artificial Intelligence for Applications.

[33] Beaufort L., Dollfus D. (2004). Automatic recognition of coccoliths by dynamical neural networks. Mar. Micropaleontol..

[34] Pedraza L.F., Hernández C.A., López D.A. (2017). A Model to Determine the Propagation Losses Based on the Integration of Hata-Okumura and Wavelet Neural Models. Int. J. Antennas Propag..

[35] Nanni L., Faldani G., Brahnam S., Bravin R., Feltrin E. (2023). Improving Foraminifera Classification Using Convolutional Neural Networks with Ensemble Learning. Signals.

[36] Huang B., Yang F., Yin M., Mo X., Zhong C. (2020). A Review of Multimodal Medical Image Fusion Techniques. Comput. Math. Methods Med..

[37] Helber P., Bischke B., Dengel A., Borth D. (2019). EuroSAT: A Novel Dataset and Deep Learning Benchmark for Land Use and Land Cover Classification. IEEE J. Sel. Top. Appl. Earth Obs. Remote Sens..

[38] Zhu X.X., Tuia D., Mou L., Xia G.S., Zhang L., Xu F., Fraundorfer F. (2017). Deep Learning in Remote Sensing: A Comprehensive Review and List of Resources. IEEE Geosci. Remote Sens. Mag..

[39] Ma L., Liu Y., Zhang X., Ye Y., Yin G., Johnson B.A. (2019). Deep learning in remote sensing applications: A meta-analysis and review. ISPRS J. Photogramm. Remote Sens..

[40] Pelletier C., Webb G.I., Petitjean F. (2019). Temporal Convolutional Neural Network for the Classification of Satellite Image Time Series. Remote Sens..

[41] Sellami A., Abbes A.B., Barra V., Farah I.R. (2020). Fused 3-D spectral-spatial deep neural networks and spectral clustering for hyperspectral image classification. Pattern Recognit. Lett..

[42] Zhang X., Ye P., Xiao G. VIFB: A visible and infrared image fusion benchmark. Proceedings of the IEEE/CVF Conference on Computer Vision and Pattern Recognition Workshops.

[43] James A.P., Dasarathy B.V. (2014). Medical image fusion: A survey of the state of the art. Inf. Fusion.

[44] Hermessi H., Mourali O., Zagrouba E. (2021). Multimodal medical image fusion review: Theoretical background and recent advances. Signal Process..

[45] Li X., Jing D., Li Y., Guo L., Han L., Xu Q., Xing M., Hu Y. (2022). Multi-Band and Polarization SAR Images Colorization Fusion. Remote Sens..

[46] Moon W.K., Lee Y.-W., Ke H.-H., Lee S.H., Huang C.-S., Chang R.-F. (2020). Computer-aided diagnosis of breast ultrasound images using ensemble learning from convolutional neural networks. Comput. Methods Programs Biomed..

[47] Maqsood S., Javed U. (2020). Multi-modal medical image fusion based on two-scale image decomposition and sparse representation. Biomed. Signal Process. Control..

[48] Ding I.-J., Zheng N.-W. (2022). CNN Deep Learning with Wavelet Image Fusion of CCD RGB-IR and Depth-Grayscale Sensor Data for Hand Gesture Intention Recognition. Sensors.

[49] Tasci E., Uluturk C., Ugur A. (2021). A voting-based ensemble deep learning method focusing on image augmentation and preprocessing variations for tuberculosis detection. Neural Comput. Appl..

[50] Mishra P., Biancolillo A., Roger J.M., Marini F., Rutledge D.N. (2020). New data preprocessing trends based on ensemble of multiple preprocessing techniques. TrAC Trends Anal. Chem..

[51] Mitra R., Marchitto T.M., Ge Q., Zhong B., Lobaton E. (2019). Foraminifera optical microscope images with labelled species and segmentation labels. PANGAEA.

[52] Kuncheva L.I. (2014). Combining Pattern Classifiers: Methods and Algorithms.

[53] LeCun Y., Boser B., Denker J.S., Henderson D., Howard R.E., Hubbard W., Jackel L.D. (1989). Backpropagation Applied to Handwritten Zip Code Recognition. Neural Comput..

[54] He K., Zhang X., Ren S., Sun J. (2016). Deep residual learning for image recognition. Proceedings of the 2016 IEEE Conference on Computer Vision and Pattern Recognition (CVPR).

[55] Huang G., Liu Z., Van Der Maaten L., Weinberger K.Q. Densely Connected Convolutional Networks. Proceedings of the IEEE Conference on Computer Vision and Pattern Recognition.

[56] Sandler M., Howard A., Zhu M., Zhmoginov A., Chen L.C. MobileNetV2: Inverted Residuals and Linear Bottlenecks. Proceedings of the 2018 IEEE/CVF Conference on Computer Vision and Pattern Recognition.

[57] Zhuang F., Qi Z., Duan K., Xi D., Zhu Y., Zhu H., Xiong H., He Q. (2021). A Comprehensive Survey on Transfer Learning. Proc. IEEE.

[58] Wang D., Zhang J., Xu M., Liu L., Wang D., Gao E., Han C., Guo H., Du B., Tao D. (2024). MTP: Advancing remote sensing foundation model via multi-task pretraining. IEEE J. Sel. Top. Appl. Earth Obs. Remote Sens..

[59] Gesmundo A. (2022). A continual development methodology for large-scale multitask dynamic ML systems. arXiv.

[60] Gesmundo A., Dean J. (2022). An evolutionary approach to dynamic introduction of tasks in large-scale multitask learning systems. arXiv.

[61] Jeevan P., Sethi A. (2024). Which Backbone to Use: A Resource-efficient Domain Specific Comparison for Computer Vision. arXiv.

[62] Zhu X.X., Hu J., Qiu C., Shi Y., Kang J., Mou L., Bagheri H., Haberle M., Hua Y., Huang R. (2020). So2Sat LCZ42: A Benchmark Data Set for the Classification of Global Local Climate Zones. IEEE Geosci. Remote. Sens. Mag..

[63] LeCun Y., Bengio Y. (1995). Convolutional networks for images, speech, and time series. Handb. Brain Theory Neural Netw..

[64] Lecun Y., Bottou L., Bengio Y., Haffner P. (1998). Gradient-based learning applied to document recognition. Proc. IEEE.

[65] Shabbir A., Ali N., Ahmed J., Zafar B., Rasheed A., Sajid M., Ahmed A., Dar S.H. (2021). Satellite and scene image classification based on transfer learning and fine tuning of ResNet50. Math. Probl. Eng..

[66] Le T.D., Ha V.N., Nguyen T.T., Eappen G., Thiruvasagam P., Garces-Socarras L.M., Chatzinotas S. (2024). Onboard Satellite Image Classification for Earth Observation: A Comparative Study of ViT Models. arXiv.

[67] Zhong X., Li H., Shen H., Gao M., Wang Z., He J. (2024). Local Climate Zone Mapping by Coupling Multilevel Features With Prior Knowledge Based on Remote Sensing Images. IEEE Trans. Geosci. Remote. Sens..

[68] Lin H., Wang H., Yin J., Yang J. (2024). Local Climate Zone Classification via Semi-Supervised Multimodal Multiscale Transformer. IEEE Trans. Geosci. Remote. Sens..

[69] He G., Dong Z., Guan J., Feng P., Jin S., Zhang X. (2023). SAR and Multi-Spectral Data Fusion for Local Climate Zone Classification with Multi-Branch Convolutional Neural Network. Remote. Sens..

[70] Dimitrovski I., Kitanovski I., Kocev D., Simidjievski N. (2023). Current trends in deep learning for Earth Observation: An open-source benchmark arena for image classification. ISPRS J. Photogramm. Remote. Sens..

[71] Qiu C., Tong X., Schmitt M., Bechtel B., Zhu X.X. (2020). Multilevel Feature Fusion-Based CNN for Local Climate Zone Classification From Sentinel-2 Images: Benchmark Results on the So2Sat LCZ42 Dataset. IEEE J. Sel. Top. Appl. Earth Obs. Remote. Sens..

[72] Ji W., Chen Y., Li K., Dai X. (2023). Multicascaded Feature Fusion-Based Deep Learning Network for Local Climate Zone Classification Based on the So2Sat LCZ42 Benchmark Dataset. IEEE J. Sel. Top. Appl. Earth Obs. Remote. Sens..

[73] Narkhede M., Mahajan S., Bartakke P., Sutaone M. (2024). Towards compressed and efficient CNN architectures via pruning. Discov. Comput..

[74] Chou H.-H., Chiu C.-T., Liao Y.-P. (2021). Cross-layer knowledge distillation with KL divergence and offline ensemble for compressing deep neural network. APSIPA Trans. Signal Inf. Process..

[75] Cheng H., Zhang M., Shi J.Q. (2024). A survey on deep neural network pruning: Taxonomy, comparison, analysis, and recommendations. IEEE Trans. Pattern Anal. Mach. Intell..

